# Explainable drug sensitivity prediction through cancer pathway enrichment

**DOI:** 10.1038/s41598-021-82612-7

**Published:** 2021-02-04

**Authors:** Yi-Ching Tang, Assaf Gottlieb

**Affiliations:** Center for Precision Health, School of Biomedical Informatics, University of Texas Health Science Center at Houston, Houston, TX 77030 USA

**Keywords:** Targeted therapies, Cancer genomics

## Abstract

Computational approaches to predict drug sensitivity can promote precision anticancer therapeutics. Generalizable and explainable models are of critical importance for translation to guide personalized treatment and are often overlooked in favor of prediction performance. Here, we propose PathDSP: a pathway-based model for drug sensitivity prediction that integrates chemical structure information with enrichment of cancer signaling pathways across drug-associated genes, gene expression, mutation and copy number variation data to predict drug response on the Genomics of Drug Sensitivity in Cancer dataset. Using a deep neural network, we outperform state-of-the-art deep learning models, while demonstrating good generalizability a separate dataset of the Cancer Cell Line Encyclopedia as well as provide explainable results, demonstrated through case studies that are in line with current knowledge. Additionally, our pathway-based model achieved a good performance when predicting unseen drugs and cells, with potential utility for drug development and for guiding individualized medicine.

## Introduction

Tailoring drugs to patients based on their genomic and environmental factors is one of the ultimate goals of precision medicine. Within precision oncology, using the molecular signatures of targeted genes has been useful for targeted therapy^1^. The use of machine learning algorithms has significantly progressed in predicting drug response by integrating genetic features and chemical structure information. Menden et al. derived multiple features from cell lines, including microsatellite instability status, mutations and copy number variations to predict drug response, and demonstrating that that chemical information improved significantly the drug models^2^. Another work, by Wang et al., used matrix factorization to predict drug response from low dimensional drug similarity space and cell line similarity space^3^ while a work by Liu et al. performed better by including drug response similarity^4^. Recently, Li et al.^5^ published a method called DeepDSC that predicts drug response by encoding gene expression features through an autoencoder and feeding the encodings together with chemical structure information into a deep neural network.

While these computational models attempt to improve performance, the focus on explainable and generalizable models remains limited. Being able to explain the model results to oncology researchers can diminish a significant barrier for translation of prediction models to clinical setting of precision oncology. In order to address this barrier, a study by Yang et al.^6^ built a Bayesian model for inferring the relation between drug target proteins and cancer signaling pathway activities. This work assumed that if a drug pathway is activated in a tumor, then the tumor cells are likely to be sensitive to drugs that target genes in that pathway. Mathematically, the drug response variable (e.g., IC50) is factorized into drug target feature and signaling pathway feature. While gaining interpretability, this approach displayed low performance.

In this study, we integrated ideas of cancer pathways with deep learning constructs to develop a pathway-based model for the prediction of drug sensitivity in cancer, called PathDSP, which both performs well and is also explainable. The rationale is that drugs exert their therapeutic effects by affecting target proteins, further signaling downstream pathways. Activation of signaling pathways thus may indicate whether cells are sensitive or resistant to a drug when the activated signaling pathways are important for cell growth or death. We integrated drug-based pathway enrichment scores across 196 cancer pathways^7^ with cell-based pathway enrichment scores in these pathways. Testing our model on the Genomics of Drug Sensitivity in Cancer (GDSC)^8^ and the Cancer Cell Line Encyclopedia (CCLE) databases^9^, we demonstrate better performance than previously published deep learning approaches while case studies show that pathway features agree with current knowledge on drugs’ mechanism of action. To the best of our knowledge, this is the first pathway-based deep neural network for drug sensitivity prediction, and it provides a flexible framework to incorporate additional pathways using prior knowledge. Demonstrating the generalizability of our approach across databases and for predicting response to new drugs and cell lines, our approach can thus be useful both for drug development and for guiding individualized medicine.

## Results

### Model selection

**Figure 1:**
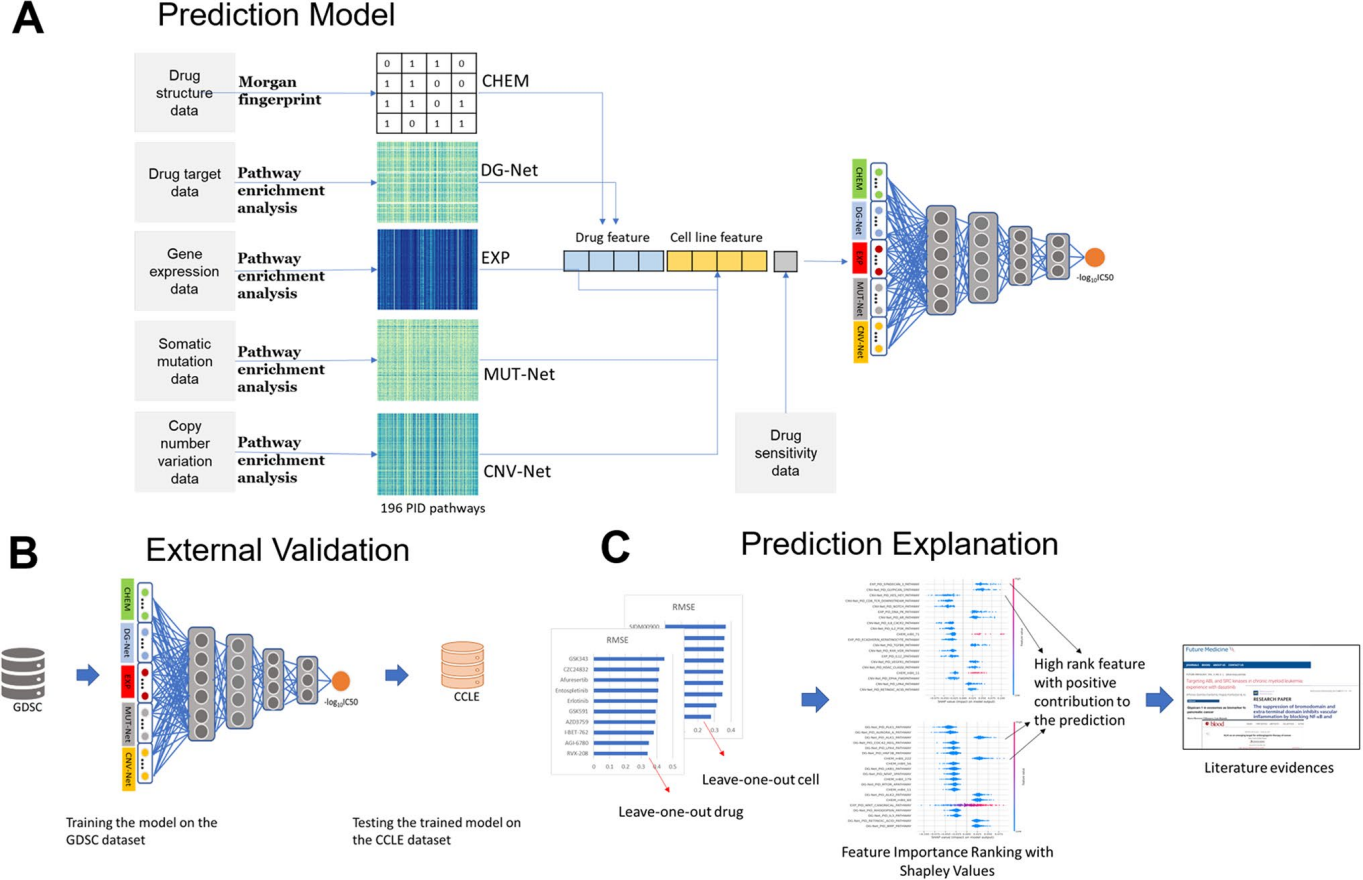
Illustration of the PathDSP method and validation plan. The chemical and cell line omics data is processed using Morgan fingerprints (chemical) and pathway enrichment (drugs targets, gene expression, mutation and CNV) is fed into FNN (**A**). The trained model on GDSC is tested on CCLE data (**B**) and SHAP scores are used to identify chemical and pathway features that contribute most to the prediction scheme (**C**).

We designed PathDSP for predicting drug response in cancer cell lines based on the rationale that cancer pathways would represent well drug therapeutic effects (Fig. 1). We predicted drug responses for 153 drugs across 319 cell lines assembled from the Genomics of Drug Sensitivity in Cancer (GDSC) with available gene expression, somatic mutation and copy number variations data (Methods). Our prediction scheme included predicting of the response values (log-transformed IC50) based on two drug-based data types and three cell line-based data types. The drug-based features include chemical structure molecular fingerprints (from here on referred as CHEM) and pathway enrichment of gene subnetwork of drug-associated genes (denoted as Drug-Gene Network, DG-Net). The cell-line-based features include pathway enrichment scores for gene expression (denoted as EXP), mutation (denoted as MUT-Net) and copy-number variation (denoted as CNV-Net) data (Methods).

We compared the performance of six machine learning algorithms, including ElasticNet, CatBoost^10^, XGBoost^11^, Random Forest^12^, Support Vector Machine (SVM)^13^, and the fully connected neural network (FNN). We use two metrics to assess the performance, mean absolute error (MAE), which is less sensitive to outliers and the root mean square error (RMSE). As our error distribution is Laplacian (*p* < 0.001 based on Laplacian test with Anderson—Darling, Watson and Kolmogorov–Smirnov statistics), MAE is more appropriate but in order to compare to previously published methods, we need the RMSE and per Chai and Draxler paper^14^, a combination of the metrics are often required to assess model performance. The result of tenfold cross validation demonstrated that FNN obtained the best performance with an MAE of 0.24 ± 0.02 and RMSE of 0.35 ± 0.02 (Table S1). Therefore, we used the FNN model throughout the study.

For the FNN model, we further compared performance of subsets of the data types. Unsurprisingly, the combination of all data types obtained the lowest MAE of 0.24 ± 0.02 and RMSE of 0.35 ± 0.02 (Table 1). Using either CHEM, EXP, MUT-Net or CNV-Net as single drug or cell-related data types performed very similar (0.31 < MAE < 0.33, 0.42 < RMSE < 0.44), while DG-Net without CHEM performed worst (MAE = 0.39, RMSE = 0.54).

**Table 1:** The MAE and RMSE of tenfold cross validation obtained by including different subsets of the data types.

Prediction scheme	Drug feature	Cell feature	MAE	RMSE	R^2^	PCC
Drug-oriented	CHEM	EXP + MUT-Net + CNV-Net	0.31	0.42	0.87	0.93
	DG-Net	EXP + MUT-Net + CNV-Net	0.39	0.54	0.8	0.9
Cell-oriented	CHEM + DG-Net	EXP	0.32	0.43	0.87	0.93
	CHEM + DG-Net	MUT-Net	0.31	0.42	0.88	0.94
	CHEM + DG-Net	CNV-Net	0.33	0.44	0.86	0.93
Reference Line	CHEM + DG-Net	EXP + MUT-Net + CNV-Net	0.24	0.35	0.92	0.96

We tested also whether the addition of external knowledge data would improve the prediction. We first examined whether adding drug class information available in GDSC as one-hot matrix improves the performance. The result showed instead a decrease in performance (MAE = 0.32, RMSE = 0.43), suggesting drug class information may overfit the model. We next tested the effect of adding combined gene essentiality scoring (CES) developed to integrate to integrate CRISPR and shRNA gene essentiality profiles with the molecular features of cancer cells^15^. We tested CES profiles in the CCLE database as it shares more drug-cell line pairs with the CES dataset than GDSC (6491 pairs vs. 2803 pairs with GDSC). Our results show that MAE remained the same (MAE = 0.22) with a minor improvement in RMSE performance (RMSEs of 0.39 vs. 0.4 without CES). Lastly, it was infeasible to perform cross-validation test for the integration of drug expression profiles from the LINCS L1000 drug-induced expression profiles^16^ due to the low overlap between the drug-cell line sets in L1000 and either GDSC or CCLE (44 drug-cell line pairs shared with GDSC and 108 drug-cell line pairs shared with CCLE). We thus used the best performing model (without drug class information and CES) for the rest of the analysis.

### Comparison with other methods

**Figure 2:**
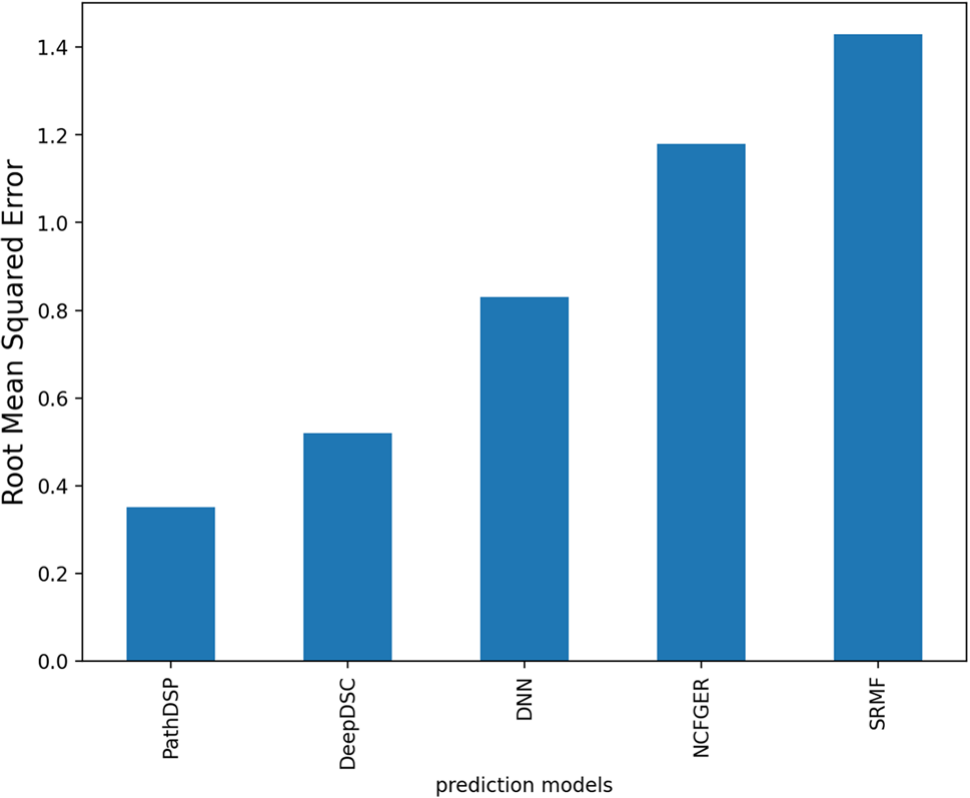
Comparison of PathDSP performance relative to four previously published methods based on RMSE.

We compared PathDSP with four previously published methods, including DNN by Menden et al., SRMF, NCFGER, and DeepDSC^2–5^. All models used the same drug response and provided only RMSE on the GDSC dataset. PathDSP outperformed these four models, where the next best performer was DeepDSC with an RMSE of 0.52 (8.5 standard deviations from our results), followed by other models with RMSE between 0.83 and 1.43 (Fig. 2).

### Prediction of new drugs and cell-lines

*In-silico* inference of drug response to a new experimental molecule would be beneficial for pharmaceutical research and drug design, reducing the high cost of large-scale drug screening tests. Predicting drug response for new cell lines, on the other hand, could be translated to clinical setting when oncologists are tasked with prioritizing treatment options for a new patient, potentially translating the cell-line genomic data to the patient’s genomic data. To address these scenarios, we performed leave-one-drug-out (LODO) and leave-one-cell-out (LOCO) by removing one drug or cell line, respectively, from training and assessing the prediction error for that missing drug or cell line (Methods). We obtained an average MAE of 0.83 ± 0.58 (RMSE of 0.98 ± 0.62) from LODO, and an average MAE of 0.45 ± 0.15 (RMSE of 0.59 ± 0.17) from LOCO. In particular, our LODO model obtained lower RMSE than DeepDSC, the only model that performed leave-one-drug-out out of the four previously compared models (1.24 ± 0.74).

Hemopoietic and solid malignancies have significantly different clinical treatments. We thus tested the performance of the model on hemopoietic cell lines (21% of the cell lines in GDSC) and solid cancer cell line separately. Cross-validation performance was similar across cell line types (MAE = 0.24, RMSE = 0.35 and MAE = 0.23, RMSE = 0.34 for haematopoietic cell lines and solid cancer cell lines, respectively). This result is much better performance than when training only on each group separately (MAE = 0.32, RMSE = 0.43 for solid cancer cell lines and MAE = 0.31, RMSE = 0.42 for haematopoietic cell lines). Conversely, in our leave-one-cell-out experiment, the performance for hemopoietic cancer cells was better (RMSE = 0.54 ± 0.13) than for solid cancer cell lines (0.60 ± 0.18).

### Generalizability of PathDSP

**Figure 3:**
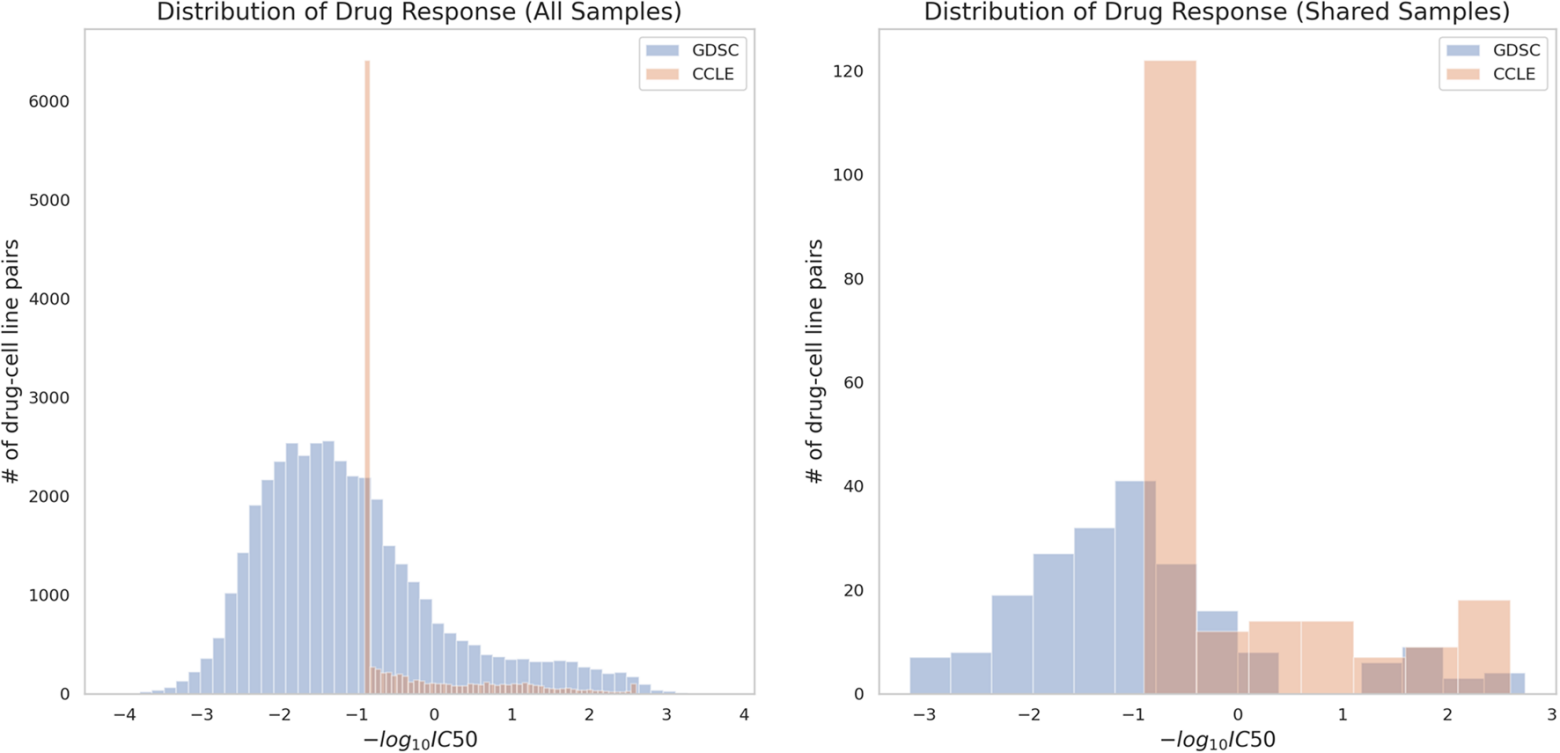
Comparison of drug response distribution (histogram of drug-cell line pairs) between the GDSC (blue) and CCLE (orange) databases.

**Figure 4:**
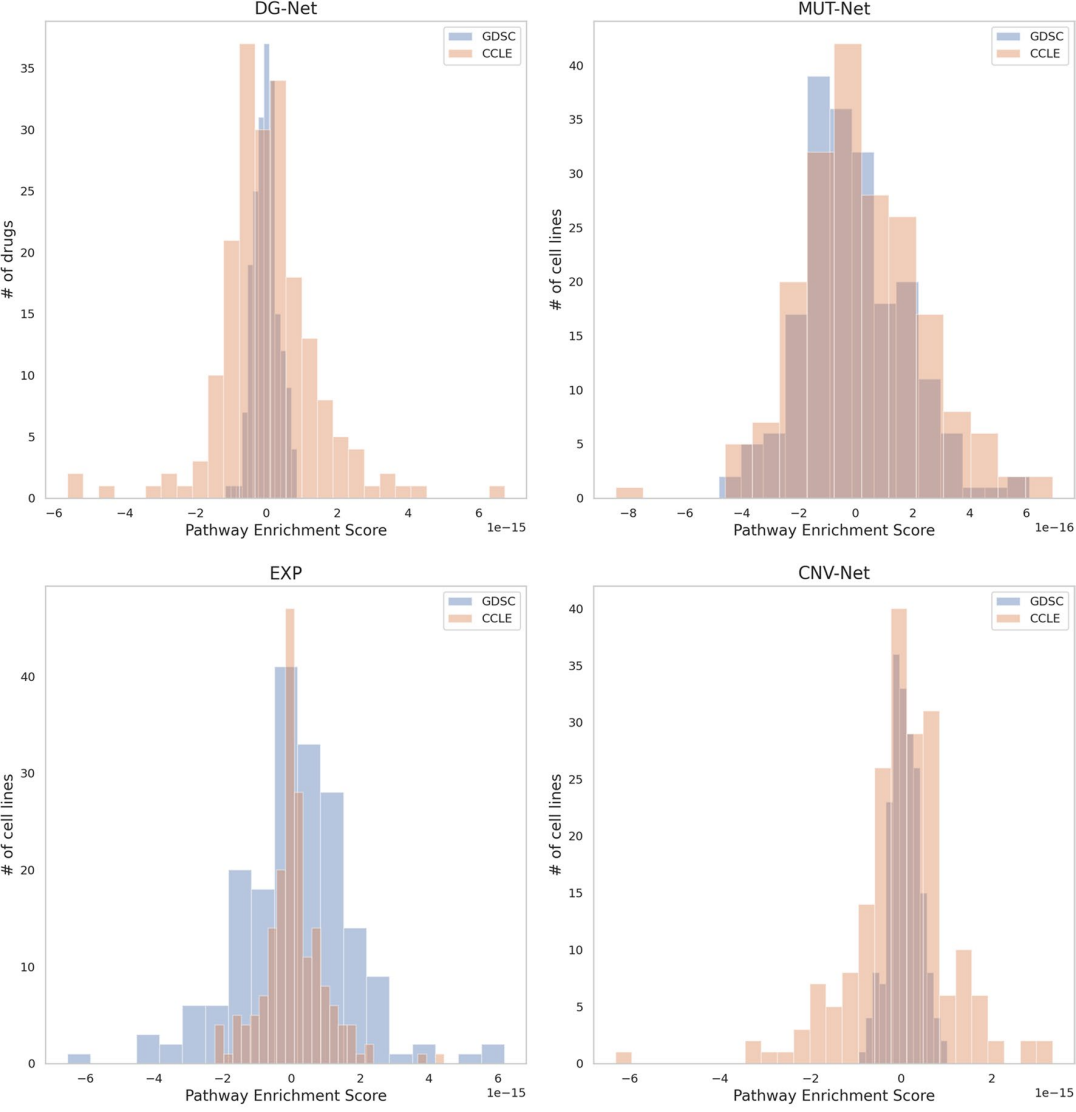
Comparison of pathway enrichment scores distribution (histogram of drugs or cell line numbers) between the GDSC (blue) and CCLE (orange) databases for DG-Net, EXP, Mut-Net and CNV-Net features.

We further tested the generalizability of PathDSP across different datasets. We applied the model trained on the GDSC dataset to an independent dataset from the CCLE^9^. PathDSP obtained an MAE of 0.93 (and RMSE of 1.15) when tested on the entire dataset of CCLE, with an MAE of 0.94 (RMSE of 1.16) when tested on drug-cell line pairs that to do exist in GDSC and an MAE of 0.74 (and RMSE of 0.95) when tested only on the set of drug-cell line pairs shared between GDSC and CCLE. Notably, the difference in the drug response measurements between the two datasets makes this task challenging. While GDSC tested a large range of IC50 values, CCLE capped their tested range at a concentration of 8 μM^17,18^, resulting in different response values of the same drug-cell line combinations across the two datasets (Figs. 3, S2). To demonstrate the difference between the datasets, we computed the expected MAE and RMSE if PathDSP had perfectly modeled the training data (GDSC). Computing the RMSE of the true response values of GDSC and testing against the response values of CCLE on the shared subset of drug-cell line pairs, the result is higher than the one we obtained with our algorithm (0.88 MAE and 1.13 RMSE vs. our predicted 0.74 MAE and 0.95 RMSE), suggesting that PathDSP is able to generalize well. Notably, despite different distributions of input features between the two datasets (e.g. expression measurements), the pathway-enriched features of PathDSP displayed more consistent patterns between two datasets (Fig. 4). These results suggest with more consistent IC50 measurements, PathDSP has the potential to obtain better performance.

### Explainability of the model

We computed Shapley values to identify important features underlying predictions. We focus on features that have positive contributions to drug response (i.e. the feature contributes to reducing IC50). The top features are distributed across the different data types, including expression, DG-Net, CNV and mutation, supporting our observation that the combination of these data types enables the good performance (figure S1). We highlight a subset of these globally important top features (i.e. features important across all drugs and cell lines in the GDSC dataset): The top feature is an enrichment of cell line expressed genes within the ADP-Ribosylation factor 3 (ARF3) pathway. The main gene of this pathway, ARF3 gene, is up-regulated in breast cancer and promotes breast cancer cell proliferation, representing a novel prognostic marker and therapeutic target for breast cancer^19^ (the GDSC dataset includes 11 breast cancer cell lines).

The second top feature is enrichment of the Histone deacetylase III (HDAC-III) pathway within the drug-associated gene network (DG-Net). HDAC inhibitors seems to be promising anti-cancer drugs^20^. Several genes within this pathway have established role in cancer, such as TP53^21,22^ and SIRT1, which is up-regulated in cancer cells and may play a critical role in tumor initiation, progression, and drug resistance by blocking senescence and apoptosis, and promoting cell growth and angiogenesis. SIRT1 inhibitors have shown promising anticancer effects in animal models of cancer^23^. The third top feature is CNV of Glypican 1 pathway, further discussed in the leave-one-cell-out specific example below. Finally, the next two features involve enrichment of the Nuclear factor kappa B (NF-κB) canonical pathway within mutation data and enrichment of Fanconi Anemia pathway by DG-Net. Indeed, mutations in the component of the core NF-κB signaling pathway have been implicated with relation to cancer^24^ and patients with Fanconi Anemia have a higher risk of cancer, particularly for acute myeloid leukemia and squamous cell carcinoma followed by ongoing work to study targeting of pathways that are synthetic lethal with loss of Fanconi Anemia pathway with Olaparib or other drugs^25^.

Next, we highlight two local feature importance predictions, one for the LODO (RVX208) and one for the LOCO scenario, demonstrating the drug- and cell line- specific explanability of PathDSP.

#### RVX208

The best performing prediction of LODO is for RVX-208 drug (apabetalone, MAE = 0.26 and RMSE = 0.34 across all cell lines). RVX-208 is a Bromodomain and Extra-Terminal domain (BET) inhibitor. BET regulates the transcriptional program and plays a role in influencing cancer pathogenesis and inflammation^26^. The Activin receptor-like kinase-1 (ALK1) pathway ranked highest out of the features with positive contribution to drug responses (Fig. S3). ALK1 is a receptor of TGF-beta type 1 receptor family and can regulate angiogenesis, which plays a critical role in the growth of cancer because solid tumors need a blood supply if they are to grow in size and tumors can actually cause this blood supply to form by stimulating angiogenesis^27^. Correspondingly, ALK1 is a well-known cancer driver which could act as a tumor suppressor or oncogene, depending on the cancer type, cell type, or ligand involved^28^ and is an emerging target for antiangiogenic therapy of cancer^29^. Additional genes in the ALK1 pathway include mitogen-activated protein kinase 1 and 3 (MAPK1 and MAPK3), where the suppression of BET inhibits vascular inflammation by blocking MAPK activation^30^.

Additional top features for RVX-208 are other pathways involved in angiogenesis, including ALK2^31^, Canonical Wnt^32^, retinoic acid^33^ and bone morphogenetic proteins (BMP)^31,34^ pathways. One way in which these pathways connect to the ALK1 pathway is via shared member genes, including the MAPK1 and MAPK3 genes that are part of both the ALK1 and the retinoic acid receptors-mediated signaling pathways; and the SMAD1/4/5 genes included in the ALK1, ALK2 and BMP pathways, where in endothelial cells ALK1 activates the Smad1/Smad5 pathway^29,35^. Another way is through cross-talk between the pathways, as in the case of canonical Wnt signaling that was found to skew TGF-β signaling in chondrocytes via the ALK1 pathway^36^.

There are three other BET inhibitors in the GDSC dataset: I-BET-762, OTX015 and JQ1. In two of the BET inhibitors, I-BET-762 and OTX015, ALK1 is also one of the top features (rank 1st in I-BET-762 and 4th in OTX015). Additionally, some of the other above mentioned pathways, including the BMP pathway (ranks 4th and 6th), the ALK2 pathway (ranks 9th and 8th) and the retinoic acid pathway only in OTX015 (rank 10). The remaining BET inhibitor in our set, JQ-1, does not display the ALK1 pathway as a top feature but displays the Canonical Wnt signaling (rank 4th) and retinoic acid (rank 14^th^) pathways. Notably, JQ-1 was only tested on breast cancer cells, while the other three BET inhibitors were tested on multiple cancer cell types, which could explain the differences in the extracted top features.

#### Chronic myelogenous leukemia cell line

Our model obtained good performance when predicting drug response for the Chronic Myelogenous Leukemia (CML) cell line (SIDM00482) in the LOCO (MAE = 0.23 and RMSE = 0.31). Based on the Shapley values, gene expression enrichment of the SYNDECAN 3 pathway and copy number variations enrichment of the Glypican 1 (GPC-1) pathway display positive contribution to drug response (Figure S4). Syndecans and glypicans are membrane-bound heparan sulphate proteoglycans (HSPGs), they act as receptors and relate to cell growth and differentiation by interacting with other growth factors^37,38^. Indeed, syndecans and glypicans have reported roles in tumorogenesis in blood cancers^39^. Specifically, GPC1 was found to be overexpressed and a biomarker of certain cancers with demonstrated ability to distinguish between healthy controls and advanced cancer patients with 100% accuracy^40^, while Syndecan-3 has not been implicated in cancer yet^38^. However, other members of these pathways include epidermal growth factor receptor (EGFR) that have been implicated with regard to activation in CML^41^, SRC Proto-Oncogene, Non-Receptor Tyrosine Kinase (SRC) that appears in both GPC-1 and syndecan-3 pathways, whose overexpression have been identified among the known mechanisms of resistance to imatinib in CML^42^ and FYN proto-oncogene, Src family tyrosine kinase that is up-regulated in CML as result of the BCR-ABL1 oncogene^43^. We note that our data includes additional five CML cell lines. Out of these five, three cell lines also have GPC-1 as one of the top important features (SIDMO00958 rank 2, SIDMO00346 rank 12 and SIDMO00962 rank 15). The other two CML cells show top pathways that are related to GPC-1, including bone morphogenetic proteins (BMP), mitogen-activated protein kinase (MAPK) and Wnt pathways. Glypicans are known to regulate BMP signaling^44^ and specifically GPC-1 and BMP expression are correlated in certain cancers like adenocarcinoma^45^, and mitogen-activated protein kinase (MAPK), where GPC-1 binds growth factors to facilitate their assembly for enhanced signaling in MAPK, among others^46^ and knockdown of GPC-1 decreased growth of ESCC cells and induced apoptosis via inhibition of MAPK signaling pathways in vitro^47^. Finally, altering GPC-1 levels modulates canonical Wnt signaling during trigeminal placode development and an in vivo role for glypicans has been demonstrated in association with Wnt signaling^48^.

These two examples demonstrate that identifying cancer pathways association with drug-associated genes can help identify potential mechanisms and possibly new targets within the pathway.

## Discussion

In this study, we presented PathDSP, a method that integrates drug and cell line information to predict drug responses of cancer cell lines. We addressed the high dimensional and heterogeneous nature of the data used in this task by mapping it to curated pathways. By using this approach, we gained improved performance over current state-of-the-art methods, improved generalizability of the models across independent datasets and provided a cancer pathway-level of explainability beyond that of the single gene or single mutation level. While we focused on manually curated cancer pathways and on specific genomics data including gene expression, mutation and copy number variation data, our framework is flexible and can seamlessly integrate additional pathways as well as other data types. We predicted IC50 values instead of reframing the problem as a binary classification problem since there is no agreed-upon threshold to determine sensitive vs. resistant cell line, owing in part to being drug- and cell-dependent^49^.

We demonstrated that integrating multiple types of data improve performance of the algorithm. Interestingly, for drug-related data types, relying only on engineered features from the drug-gene-network without chemical structure information obtained the worst performance, but the combination of the two improved performances over only one of them. Similarly, with regard to cell line specific data types, each of the individual cell-line data, i.e. gene expression, mutation and copy number variation, gain similar but reduced performance relative to their combination. Nevertheless, the reduction in performance in this case is smaller, holding a promise that our method could be applied even within clinical settings with only a subset of the data types measured. While external data, such as gene essentiality score (CES) or the L1000 gene expression profiles for drugs holds promise to further improve the models and their biological interpretation, the current low overlap between these datasets and the drug sensitivity datasets of GDSC and CCLE limits the integration of these datasets.

The incompleteness of drug target data poses a potential limitation on good performance of PathDSP. In order to address that, we expanded the list of drug targets to include drug-associated genes from curated external databases and additional functionally-related genes by searching close neighbors of drug target genes within a protein–protein interaction network, potentially capturing also cross-talking pathways. As we demonstrate, the differences in response value measurements (IC50) across databases pose a limitation on the generalizability of methods designed to predict the response value, including PathDSP. However, we managed to improve the generalizability beyond the expected theoretical difference between the datasets (MAE = 0.74 and RMSE of 0.95 vs. MAE = 0.88 and RMSE = 1.13 theoretical), demonstrating good generalization capabilities, which would be critical in translating this method into clinical setting.

Last, while pathway activity may be helpful in predicting drug response differences, biological pathway usually consists of several genes responsible for diverse functionality. Thus, more fine-tuned, drug and cell-line specific, experiments are necessary in order to pinpoint which gene(s) may be the ones most associated with a drug’s sensitivity or resistance. Additionally, while the explainable associations are good predictors, they are not necessarily causal, a relationship that would require additional experiments to establish.

## Conclusion

Our developed method of pathway-based deep neural network for drug sensitivity prediction demonstrated improved performance, generalizability and exemplified explainability. Given the flexibility of this approach, we believe it provides another means in which the roles of pathways in drug response for cancers can be evaluated and to provide another steppingstone towards cancer precision medicine.

## Methods

### Data

Drug sensitivity data, cell-line gene expression, somatic mutation and copy number variation data for 319 cancer cell lines and 153 drugs was downloaded from Genomics of Drug Sensitivity in Cancer (GDSC downloaded from https://www.cancerrxgene.org/downloads/bulk_download.) Drug sensitivity data of 24 drugs and 478 cancer cell lines from the Cancer Cell Line Encyclopedia (CCLE) were downloaded through the DepMap portal (https://depmap.org/portal/download/, release version: public 20Q1), which also include gene expression, mutation and copy number variation data for those cancer cell lines. Primary target data was downloaded from GDSC, and PID pathways were downloaded from MSigDB^50^. Protein–protein network was downloaded from STRING database^51^, including protein interactions, co-expression and text-mined interactions.

### Feature engineering and normalization

#### Gene expression data

Gene expression data were measured by transcripts per million (TPM) and log-transformed. We imputed with mean for the rest of missing values. Enrichment score (ES) of each PID pathway in each cell line was calculated using the single-sample Gene Set Enrichment (ssGSEA) algorithm^52^ through GSEApy (https://gseapy.readthedocs.io/en/master/gseapy_example.html). We ran permutation test for 1000 times and normalized ES scores by the size of gene set to obtain normalized ES (i.e., NES). We used the resulting pathway enrichment matrix with size of 319 cancer cell lines by 196 pathways as the gene expression feature (EXP).

### Somatic mutation data

Mutation data are in long form, in which each row consists of a cancer cell line and its mutated gene name. We collected all mutations for each cell line to perform network-based pathway enrichment analysis by the NetPEA algorithm^53^, which calculates an enrichment score by measuring the closeness of pathway genes to a given gene set within a protein–protein interaction (PPI) network. We implemented the algorithm with some modification. The method is summarized as follows: First, both mutation gene and pathway genes were mapped to STRING PPI network^51^. For each cell, its mutation gene set is then used as the restart nodes to diffuse information through edges to their neighbors within the PPI by the Random Walk with Restart approach. For each pathway, a similarity score between the mutation gene set to the pathway genes is calculated by averaging all values on the pathway genes, where the node value represents the probability of being revisited (i.e., the closeness to the restart nodes). We modified the similarity score by multiplying each gene score with its gene expression value within the cell line, forming a cell-specific gene co-expression-mutation network. Then, a permutation test was performed 1000 times by randomly selecting the same number of genes, resulting in 1000 association scores as the background for the pathway. Pathway significance was normalized using z-score, resulting in pathway enrichment matrix with size of 319 cancer cell lines by 196 pathways for the mutation feature (MUT-Net).

### Copy number variation data

Copy number variation data were represented by the GISTIC (Genomic Identification of Significant Targets in Cancer) score comprising of − 2 (deletion), − 1 (loss), 0 (diploid), 1 (gain), and 2 (amplification), genes with GISTIC score of 0 were excluded. For each cell line, we collected a set of genes with copy number variations to calculate pathway enrichment score using the same procedure used for mutation data, resulting in the pathway enrichment matrix with size of 319 cancer cell lines by 196 pathways for the copy number variation feature (CNV-Net).

### Drug gene network data

The primary target genes were provided in the GDSC data, we further expanded the target genes by two approaches. First, we obtained off-target genes from the DGIdb version 3 database^54^, a webserver collected curated drug-gene interaction data from literature. Second, we used the expanded gene list to find its neighbors within the STRING PPI network^51^, which increases the number of related gene per drug from 4.71 to 876.78 on average. For each drug, we performed pathway enrichment analysis against 196 PID pathways^7^ using its expanded target gene list. We used the original NetPEA approach described by Liu et al.^53^. i.e., we did not aggregate gene expression value to the node like we did for mutation and copy number variation data. As a result, we obtained the pathway enrichment scores in drug targets of size 144 drugs by 196 pathways for the drug gene network feature (DG-Net), excluding nine drug without target information or the target was missing from the PPI network.

### Chemical structure data

We retrieved canonical SMILE strings by searching the PubChem database^55^ with the open-source Python API, PubChemPy (https://pubchempy.readthedocs.io/en/latest/). We then converted SMILE strings into Morgan fingerprint with the open-source cheminformatics toolkit RDKit (http://www.rdkit.org), generating the matrix of 153 drugs by 256 molecular bits for the chemical structure feature (CHEM).

### Model fitting

We created a fully connected neural network (FNN), following an architecture suggested by Li et al.^5^ using Pytorch^56^, parameters used is listed in the Table S2. We performed tenfold cross validation for the experiments in this study with early stopping applied to avoid overfitting, repeated five times with different splits for the data for robustness verification and for computing the standard deviation between runs. Before feeding into the FNN model, we normalized our features using z-score. For the experiment of leave-one-drug-out, we took out one drug from training each time (with all its cell-lines), and for the experiment of leave-one-cell-line-out, we took out one cell-line from training (with all its drugs). We used RMSE to estimate generalization error of the model. For compared machine learning algorithms besides FNN, we used scikit-learn API^57^ for the other models, including the XGBoost algorithm with hyperparameter tuning and early stopping.

### Generalization assessment against CCLE

We tested the generalizability of PathDSP by training on GDSC and applying the trained model to the CCLE dataset. We then calculated MAE and RMSE for all samples and additionally for six drugs and 35 cancer cell line pairs shared between the CCLE and GDSC datasets.

### Feature importance

Feature importance was measured as the amount of contribution each feature makes to the prediction value. We used the Shapley value^58^ to estimate feature importance, which is measured by comparing the prediction value obtained with the feature and without it^59^. The python library SHapley Additive exPlanations (SHAP)^60^ was used to obtain global feature importance and visualization of feature importance at the local level. If a feature X has a positive shapely value, it indicates that feature X contributes to a higher predicted value, and vice-versa. Thus, in this study, it is interpreted as the feature X increases/decreases drug responses to drug Y (i.e. increase in −log(IC50), which means decrease in IC50).

## Supplementary Information

Supplementary Information.

## Data Availability

All relevant data used in this study is publicly available and detailed in the data section of the manuscript.

## Code availability

PathDSP code is available at: https://github.com/TangYiChing/PathDSP
