# Supplementary material for: Explainable drug sensitivity prediction through cancer pathway enrichment
Source: Sci Rep. 2021 Feb 4;11:3128. doi: 10.1038/s41598-021-82612-7 (PMC7862690; doi:10.1038/s41598-021-82612-7)

**PathDSP: Explainable Drug Sensitivity Prediction through Cancer Pathway Enrichment**

Yi-Ching Tang^1^ and Assaf Gottlieb^1^

^1^Center for precision health, School of Biomedical informatics, University of Texas Health Science Center at Houston, Houston, TX, 77030

## Supplementary material

Table S1. Mean absolute error and root mean square error of various standard machine learning algorithms trained with CHEM + DG-Net + EXP + MUT-Net + CNV-Net feature combination.

| Model | MAE on the GDSC dataset | RMSE on the GDSC dataset |
| --- | --- | --- |
| FNN | 0.24 | 0.35 |
| CatBoost | 0.34 | 0.46 |
| XGBoost | 0.34 | 0.46 |
| ElasticNet | 0.38 | 0.51 |
| SVM | 0.54 | 0.68 |
| Random Forest | 0.54 | 0.7 |

* FNN: fully connected neural network, SVM: support vector machine

Table S2. Parameters for the fully connected neural network.

| Hyperparameter | Value |
| --- | --- |
| Hidden units | [1000, 800, 500, 100] |
| Initial weight | He normal initializer |
| Learning rate | 0.0004 |
| Drop out | 0.1 |
| Early stopping | 30 |
| Weight clip | 5 |
| Optimization function | Ada Max |
| Activation function | ELU |
| Epoch | 800 |

Figure S1. The Barplot of feature importance for the best performing model from 10-fold cross validation.


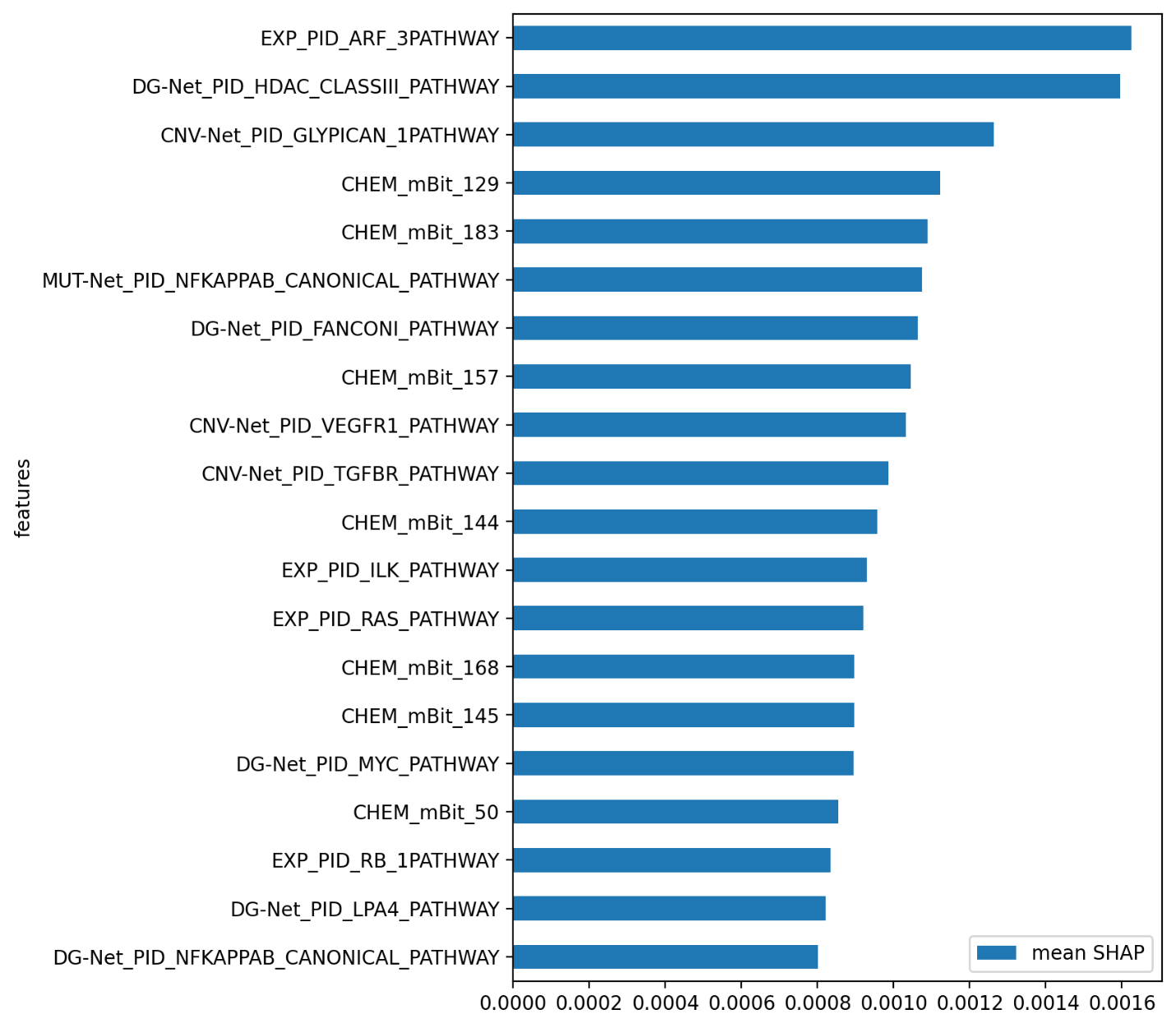


Figure S2. Scatter plot of drug response (-logIC50) for the shared samples between GDSC and CCLE (Pearson correlation coefficient, r=0.78, p~0).


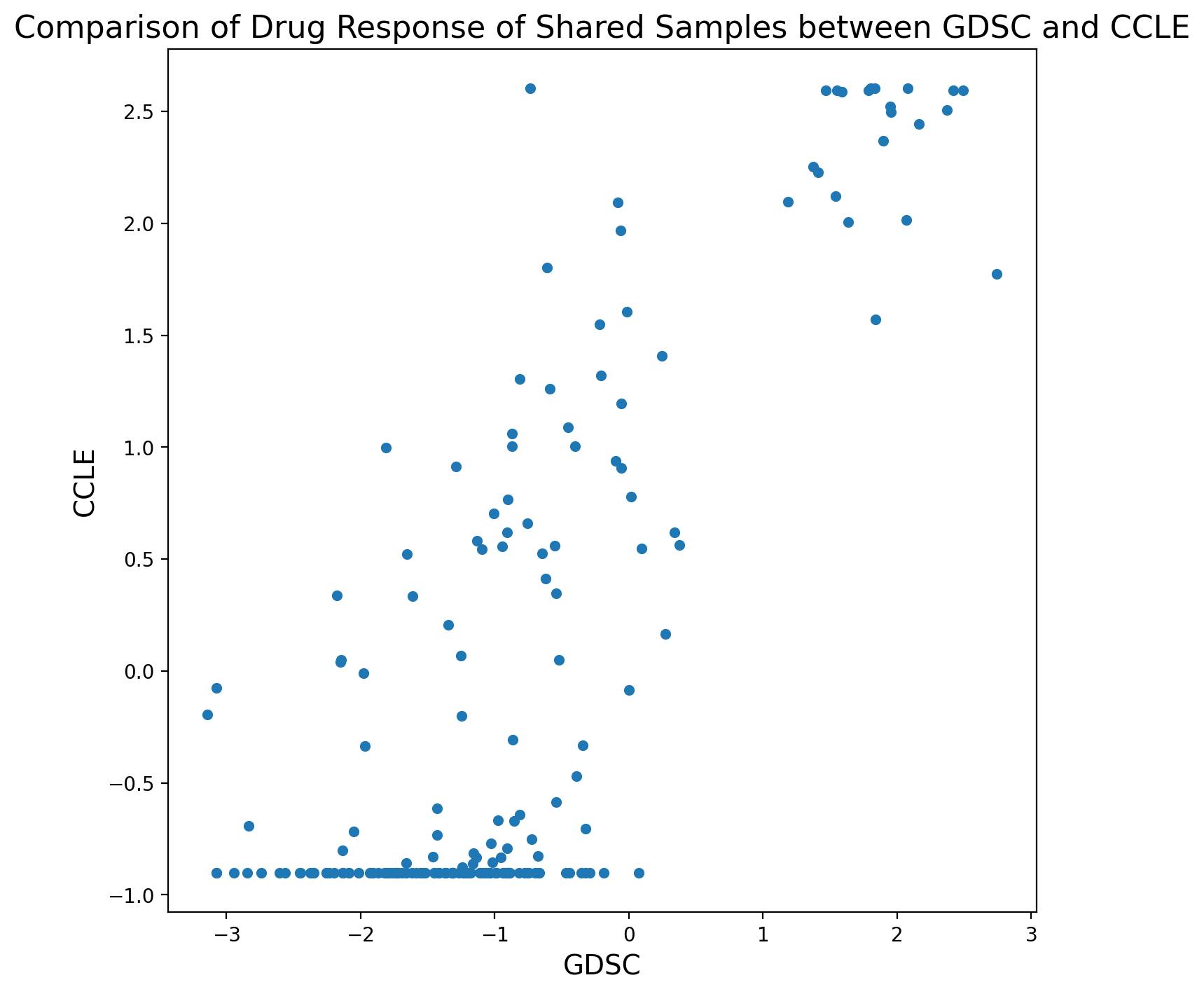


Figure S3. Summary plot of feature importance for the prediction of RVX-208 drug.


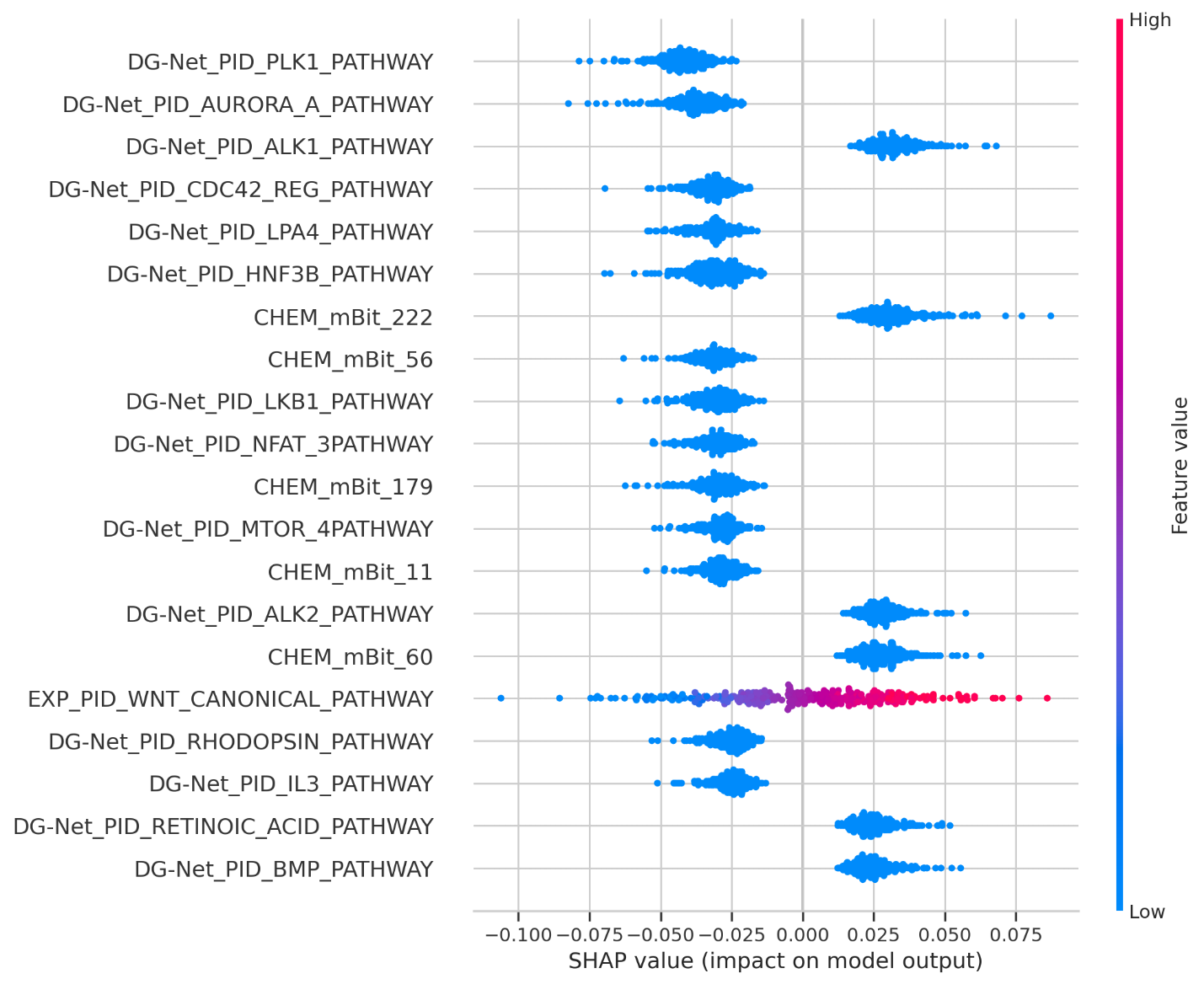


Figure S4. Summary plot of feature importance for the prediction of Chronic Myelogenous Leukemia cell line (SIDM00482).


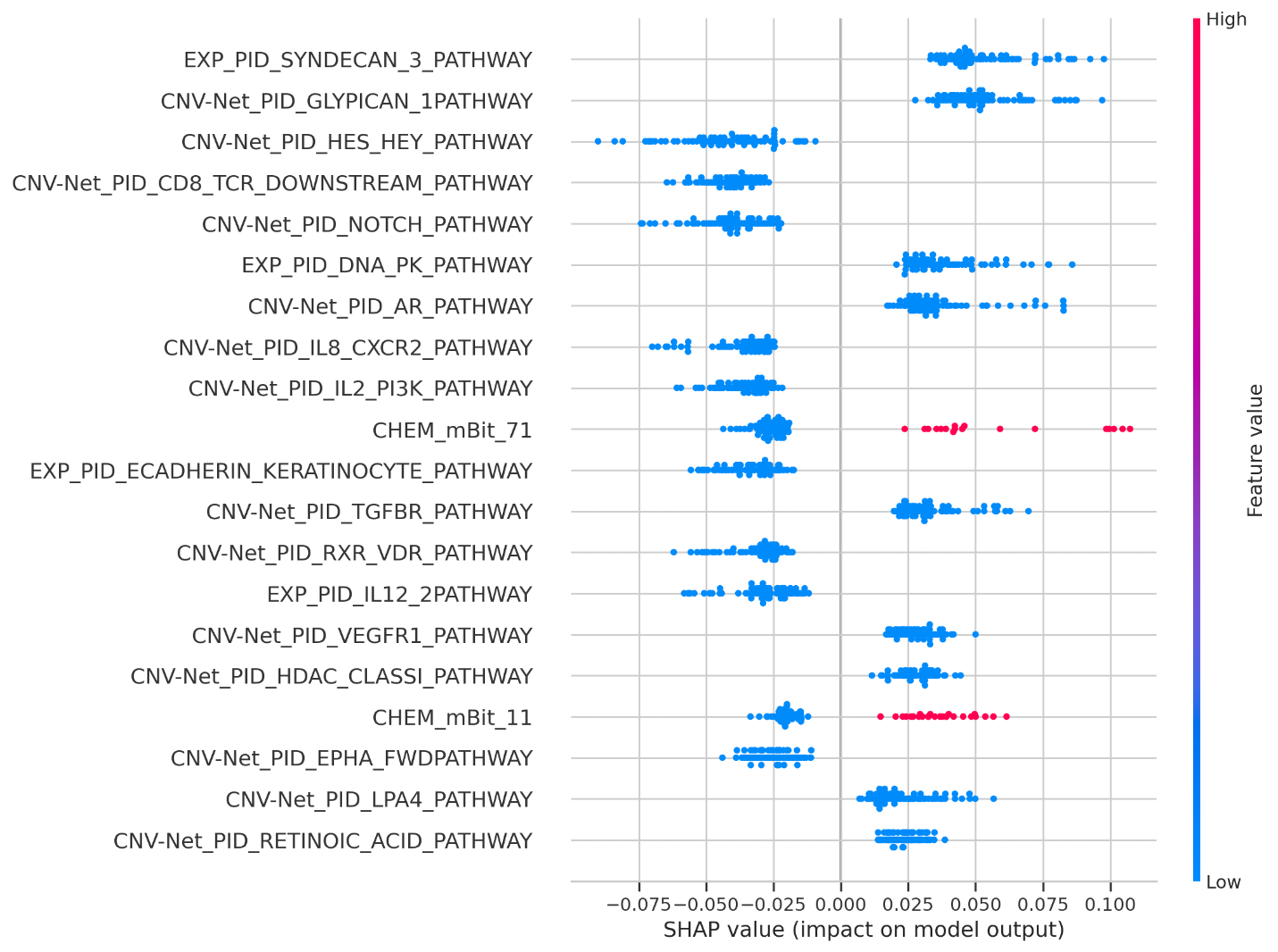

Supplement: Supplementary file 1 — Supplementary Information. [file 41598_2021_82612_MOESM1_ESM.docx]
